# Progress in Cancer

**Published:** 1902-03-15

**Authors:** 


### PROGRESS IN CANCER.

The present treatment of inoperable cancers is well summed up by Alfred Cooper [1] thus:—

1. Inoculation with the streptococcus of erysipelas.
2. Subcutaneous injection of Coley's fluid.
3. Injection of Vlaieff's anti-cancerous serum.
4. Oöphorectomy in carcinoma mammæ (Beatson).
5. Thyroid feeding (Beatson).
6. Lymph-gland extract (Snow).
7. Injection of various irritating substances:
   Parenchymatous injection of acetic acid, alcohol, methyl violet, venom of cobra di capello.
   Artificially produced suppuration by
   (1) Oil of turpentine.
   (2) Arsenious acid.
   (3) Calcium carbide.
8. Electricity.
9. Drugs—chelidonium magus (celandine) $\frac{1}{2}$-5 gr. in 24 hours, and morphia in increasing doses.

He considers that in all cases of inoperable sarcoma the patients should have the choice of having Coley's fluid injected, since some cases have been cured. In inoperable carcinoma mammæ in women of about 40 years of age, in whom the menopause has not occurred, the operation of oöphorectomy with or without thyroid feeding should be offered. That in cases of inoperable rodent ulcer and in superficial malignant ulcerations the Roentgen rays give a good hope of improvement. And that in cases where these or other methods are declined or inapplicable, the internal administration of celandine is worthy of trial. Eventually morphia should be pushed without hesitation. But in all these methods the patient should not be buoyed up by the magnification of the chances of success, but should be left to decide for or against the discomforts of the treatment.

To this list yet another has been added by John Holden Webb,[2] of Melbourne, who has injected soap solution locally or generally in inoperable cancers with some success. Speaking of the nature of cancer, he points out that the area of the Victoria (New South Wales) goldfields is all volcanic plateau, with no alluvial soil or real river, and yet cancer is very prevalent. He asks whether the cause, as in thyroid disease, may not be found in water, perhaps because of its want of mineral elements. Referring to the fact that a large number of persons with gall-stones die of malignant disease, while those with stone in the kidney do not; and also to the rapid exhaustion in cancerous patients, and the great offensiveness of them which is not due to sloughing, otherwise ether would remove it, he considered that perhaps in cancer some secretion was lost from the cells, and thought that this might be cholesterine. Acting upon this idea, he injected cholesterine in soap solution and animal gum obtained from bile, giving, at the same time, thyroid extract by the mouth. Under this treatment a recurrent epithelioma of the face was [illegible]. But the cholesterine was found to be only in suspension and not in solution, and on taking more trouble to dissolve it he injected the solution into a second case, only to find that the tumour grew very rapidly and the patient died. In the next case he injected soap solution with suspended cholesterine, and gave thyroid by the mouth. It was a case of cancer of the breast, with glands pressing on the nerves of the axilla. The whole disappeared in less than six weeks, and the firm cicatricial tissue dissolved in its turn, leaving a little retraction of the nipple only. Fifteen months later it recurred; injections were again made, and a large slough separated from a foul malignant ulcer, as big as half-a-crown, with indurated base. This again disappeared under soap and thyroid.

A rodent ulcer of 11 years' duration next disappeared under the same treatment of thyroid and soap solution with cholesterine, but failure was met with in a tumour of the breast. At this time he lost his belief in the cholesterine, and considered that the soap was the beneficial agent. An epithelioma of the hand was treated with success, and an epithelioma of the tongue was treated without thyroid, owing to cardiac trouble. In the latter improvement occurred, but the general exhaustion was such that the patient died, without, however, pain or offensive odour; and the same is claimed for a very advanced case where the tongue, lower jaw, palate and tonsil had been removed. Hitherto he has met with failure in all cases of malignant disease starting from bone. The treatment is not without its risks. One case died 24 hours after a dose of ʒii. of soap solution given at once. The solution should be just thick enough to blow bubbles with, boiled and injected warm. The local reaction is tenderness without suppuration. The dose should be only a drachm at a time. The author recommends Allen and Hanbury's superfatted soaps, but holds that yellow bar will give good results. It should be dissolved in distilled water and strained through closely woven calico. He advances the view that malignancy is due to the crystallisation of cholesterine from the living cells: and to this cause he attributes the offensiveness of cancer and the clearing up of this by means of ether. He regards myxœdema as a cancer of the fibre elements and is controlled by thyroid administration. Carcinoma is a cancer of the fibre and cell elements, and is to be controlled by the exhibition of both thyroid and soap.

Evidence for and against the parasitic aetiology of cancer is always forthcoming, and as convincing arguments and experiments seem to be adduced on either side, Borrel [3] (Annales de l'Institut Pasteur) sums up the evidence upon which the parasitic theory is based, and concludes that there is no ground of support for any of these theories. He brings forward evidence to show that the so-called "Blastomycetes" are changes in the attractive sphere and centrosome of carcinoma cells. He shows that such changes are normal in the development of the spermatozoon in the guinea-pig's testicle: a collection of portions of the sphere at times occurring about each of several centrosomes producing appearances like those seen in the "spore-cyst" of the believers in the parasitic theory. The blastomycetes of some observers in culture experiments he believes to be accidental infections.

Vlaieff [4] on the other hand holds that certain blastomycetes are the infective agents in carcinomatous tumours. He has isolated similar organisms and has obtained granulomatous tumours by injection into guinea-pigs. He has also obtained a serum from inoculated animals of different species, which will render rats and monkeys immune to the growths caused by the blastomycetes; those injected living longer than those not injected with the serum. In rats and monkeys adenomata of cylindrical epithelium developed, but if the injection of the serum was started early enough no growths developed. Vlaieff has used the serum in 60 cases of human carcinomata of all kinds, and concludes that if administered early while the growth is local, and before ulceration has occurred, it is capable of a curative effect. In more advanced cases, while no curative effect was found, it seemed to check advancement and to improve the general condition. The serum was obtained from asses and geese inoculated by cultures of the blastomycetes obtained from human cancers. He uses a dose of 5 to 10 c.cm. and there is a local reaction of swelling, and a temporary enlargement of the cancer itself lasting 48 hours. Small growths appear to undergo cystic degeneration, the larger to be impeded in their extension.

Loeb [5] has successfully transplanted portions of a sarcoma from the thyroid gland of a white rat, but has failed to produce the disease in animals of other species.

Levin [6] has repeated Lack's classical experiments in attempting to graft normal ovarian cells into other parts of the body, but examining the animals after four months has failed to find growths of any kind.

Speaking of the prophylaxis of carcinoma Keetley [7] looks for a possible agent of infection in unsterilised milk, butter and cheese. He urges upon all that no irritation, inflammation, or suppuration should be allowed to become chronic, that women should never allow anything but clean, smooth linen, cotton, or silk to come in contact with the nipples; they should be washed with soap and water, but not handled otherwise, and after suckling the strictest cleanliness and protection with clean, dry coverings should be practised.

1 Lancet, Oct. 12, 1901. 2 Ibid. 3 Amer. Jour. of Med. Sci., Sept. 4 Brit. Med. Jour., Sept. 14. 5 Brit. Med. Jour., Oct. 19. 6 Ibid. 7 Lancet, Aug. 31.

